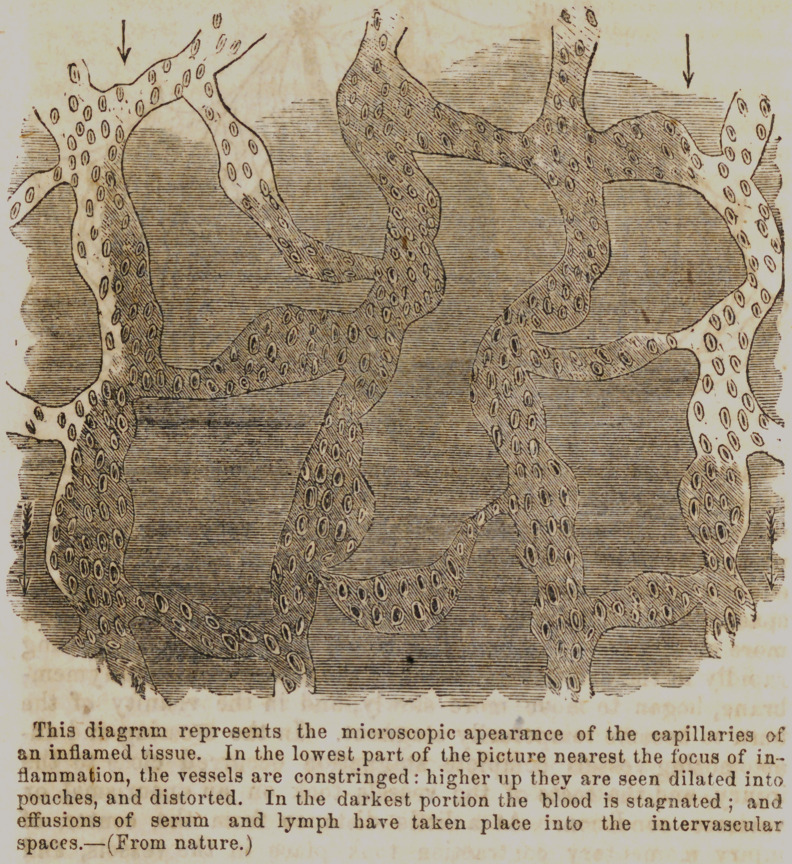# Inflammation

**Published:** 1858-08

**Authors:** C. C. Dills


### INFLAMMATION.

BY C. C. DILLS.

Inflammation may be defined to be a deviation from the healthy physiological condition of a part, occasioned by a perverted condition of the blood or blood vessels, and attended with pain, heat, redness and swelling; with the action of the part, partly increased or partly diminished; accompanied by general febrile action.

Simple as the process of inflammation is of late shown to be; yet, it has given rise to much discussion and a variety of opinions: for instance, some denominate everything inflammation, from the simple turgesence of a part, to the active ravages of supuration.

I propose to draw a line of demarcation, as far as it is practicable, between the two. It is true we may have some, or all of the foregoing symptoms enumerated, and yet not have inflammation. The blush upon the cheek, from mental excitement, or the hectic spot is entirely different from true inflammation. The simple physiological turgescence of the salivary glands during mastication, and of the stomach during digestion, and the liver during the secretion of bile ; differ very widely from that of gastritis and hipatitus. In the one case it is not at variance with health, while in the other it is a perverted action resulting in the arrest of secretion, and structural change, and disintegration of the organ.

The inflammation which closes a flesh wound, is far short of that which renders its lips, swollen pouring out a copious perulent discharge, yet they are both specifically inflammation, differing only in degree. Notwithstanding, there is every shade and degree of inflammation imaginable, yet from health to inflammation is not one step at once attained, but a gradual transition, passing through a series of stages, as we shall hereafter try to present, occupying different periods for its accomplishment, according to circumstances. For this purpose we propose to divide the process of inflammation into four stages :

1st. Simple vascular excitement.

2nd. Determination.

3rd Active congestion.

4th. And lastly, true inflammation.

If an irritant be applied to any part of a living structure, supplied with nervous sensibility, when all its functions are in healthy play, the nerves being more impressible, the immediate result is pain. This sensation is immediately transmitted to the sensorium, or great nervous center, from thence by a reflex action, to the part thus affected. The contractile fibre is thus impressed, and more particularly that which exists in the coats of the vessels, causing a diminution in their calibre. The time occupied to accomplish this result will be found to vary according to circumstances. This process begins with simple vascular excitement, and it is in this as in all other cases, the natural law governing contractile tissue is brought into play, and as a consequence, as has been stated, as before the calibre of the capillary vessels, as well as those of the adjacent arterial and venous ramifications are constricted, consequently the blood is forced through them with greater velocity than is natural, until the irritability is exhausted, then follows reaction and relaxation, and when the cause is persistent paralysis or a passive condition, which enables them to yield to the increased flow of blood to the part, till the vessels, which in a normal condition could not receive more than a single file of these red corpuscles, now very readily receive two or three.

These vessels, up to this time were not perceptible to the unassisted eye, but are now traced quite readily.

And now we have reached the Second Stage or Determination.

As the capacity of vessels gradually increase, the flow of blood to the part is greatly augmented, but very soon the flow of blood through the part becomes more and more tardy, and this condition of things favors effusion of the watery elements of the blood. The material effused may be wholly serous, or the liquor sanguineous may be found in interstitial spaces.

In the second stage of the inflammatory process, there is an abnormal amount of blood in the part, and as a consequence there is a marked tendency to exudation, partly serous and partly of a plastic character, differing but very little if at all from the ordinary liquor sanguineous.

If the exciting cause be now removed, the part may regain its former condition without advancing, but on the contrary it advances and brings us to the

Third Stage, or that of Active Congestion. The arterial portion of the vascular system, is the active agent in this stage of the disease. These are not only increased in size, but their contractions or pulsations are accelerated, hence it follows that there is an augmented flow of blood to the part till the capillary and minute arteries begin to fail under their burden—prior to this they were dilated, 'tis true, but enabled to control the circulation. This dilated condition of the blood vessels, and change in the constituents of the blood, both tend to retard the flow of blood. The red corpuscles cling to each other, and the white or lymph corpuscles are increased either relatively or locally, with a strong tendency to adhere, not only to one another, but also to the sides of the blood vessels, thereby very much impeding the flow of blood through them.

Exudation is very much favored by this condition of things, hence, it follows that the effusion is greater in this than in the preceding stage, and it also differs in character, being chiefly liquor sanguinis, altered from the healthy standard. The febrine is increased, not only in quantity but in plasticity. Vital attraction has been given as the prime cause—the over distention of the vessels, their attenuation and relaxation are no doubt the great cause. There is an approximation in this stage to hypertrophy, yet it falls one step short of that end. The plastic material is abundant, yet there is a want of formative power or appropriation—in other words there is a superabundance of nutritient material, or more supplied than can be properly appropriated to the wants of the system.

We have now come to the Fourth Stage, or True Inflammation. We have now reached a point where all the phenomena, which result from a change in the blood has been accomplished. The capillary system is paralysed, with their coats spongy, softened and impaired, being themselves the subject of structural change. The circulation is languid even unto stagnation, and this is greatly favored by the vicidity of the blood, by increased vital attraction between the corpuscles, as well as their proclivity to adhere to the parities of the vessels. The attenuated and softened capillaries give way, and the lesion blood is poured out en masse. Suppuration is in process, by extra vascular degeneration of the febrinous exudation. This infiltrated liquor sanguineous softening, and the parench is broken up, and these mix with the contents of the vessels, and then we have formed semi-purulent matter. We have now reached one of the most destructive elements in the process of inflammation. The entire part is greatly perverted, and wholly inimical to repair or continuance of normal functions.

In the immediate vicinity of an inflamed part, the circulation is stagnant, or at best sluggish, and the vessels adjacent have forced upon them increased action, till they become over-burthened, and at last are involved in the vortex of diseased action. Thus inflammation travels from structure to structure, till the entire system becomes involved, or the force of the disease becomes exhausted, or death supervenes. The alteration of the blood begins in the 2nd and is completed in the 3rd stage.

Such we conceive to be true inflammation. The blood is metamorphosed, with a strong tendency to stagnation. The blood vessels are but passive tubes, their coats spongy, soft and lacerable, unusually active. The colateral circulation unusually active, copious watery effusions, and extraras of blood by lesion of the capillary coats, function of the part wholly perverted, structure changed, suppuration in progress, and rapid disintegration of the part involved—nothing healthy, but on the contrary, all essentially diseased.

We will now very briefly consider some of the local symptoms of inflammation.

*Pain* which accompanies inflammation, is an altered or perverted condition of the nerves of sensibility. Stretching and compression of the nervous fillaments in bony canals or in fibrous structures, very greatly augment the pain. Redness is a symptom very various, dependent upon circumstances. The degree of redness varies according to the natural vascularity of the part, and the amount of active congestion attending the disease. The tint also varies according to the character and accompaniments of the action. A bright arterial redness is exhibited, by the acute and asthenic; the chronic asthenic are denoted by a dark, venous or purple hue. Heat, attendant billiary derangement gives a yellowish red, as in bilious erysipelas.

*Heat* is seldom, if ever, absent in inflammation, yet its presence does not prove that inflammation exists.

The presence of this symptom is easily explained when we consider that the great source of animal heat is to be found in the chemical changes affected upon the blood in the capillaries—changes which during the inflammatory process, are evidently carried on with great rapidity and energy, though in a perverted manner. The heat of the inflammatory process is, therefore, partly actual, as ascertained by the touch or thermometer, and partly the result of perverted nervous action—estimated only by the patient. The inflammatory heat, like the redness which is so closely connected with it, is seldom very transitory, and this is an important characteristic mark. Blushing brings heat as well as color, but both are evanescent.

*Swelling* may be caused by an undue amount of blood in the part, but the inflammatory swelling is mainly caused by the escape of a portion of the vascular contents into the intervascular spaces. This is followed by softening of the effused material, as well as the coats of the vessels, and as a result the part becomes much enlarged, and the process of inflammation is thus completed.

This being a subject of great and universal interest, and having some very fine cuts, illustrative of the various inflammatory conditions, we take the liberty to give as follows, some of the *Phenomena of Inflammation* as given by Prof. Howe in the College Journal.

"It is found by various examinations conducted with the microscope, and under the most favorable circumstances, that, when a stimulus or irritating agent is applied to a living animal tissue, the vessels, especially the arteries, take on unusual contractility, which lasts for a period varying according to the strength of the stimulus and the nervous susceptibility of the part, and then they begin to dilate, and continue in that condition until the size of the vessels is so much increased that they will transmit two or three times as much blood as they ordinarily do. Although this is contrary to the commonly received opinion, which is that the vessels take on an increase of calibre and activity as soon as the stimulus is applied, the fact that contraction takes place is nevertheless sustained by the later observations of Weber, Lebert, Paget, Virchow, Wharton Jones and others. * * * *

There are, however, certain structures when affected by inflammatory action that will not admit of all the changes referred to. These are the non-vascular tissues, as the cornea, the articular cartilages, and even the serous membranes ; yet in the immediate vicinity of such structures the blood vessels from which is derived the nutritive material and through which the ordinary vital changes take place in a state of health, are influenced in the same manner as in other inflamed structures ; and it would be unreasonable to call the process leading to ulceration of the cornea, or diseased articular cartilages, by a name different from that which we give to the coincident and similarly excited process in the other tissues.

To demonstrate some of the changes that take place in an inflamed part, Mr. Hunter froze one of the ears of a rabbit and allowed it to thaw in a way that was followed by active inflammation. When the abnormal action was at its height the animal was killed, and the head having been injected the ears were removed. These being spread out and examined, it was observed that certain changes had taken place in the one that had been inflamed. From the increased size of the vessels in the ear which had been frozen, it was inferred that inflammation increases the calibre of the vessels primarily, as well as for a season ; and from his imperfect experiment and ill-devised hypothesis many fallacies crept into the general ideas of the inflammatory process. The effect of the cold upon the ear should have been observed from the commencement of the freezing process, as well as the effect of the inflammatory element when it set in. Instead of which only the secondary effects were observed.

In addition to the experiment of Mr. Hunter, several others have been performed upon the web of a frog's foot and the delicate membranes of other cold blooded animals, for the purpose of eliciting a right understanding of the nature and manifestations of inflammatory action. Mr. Paget, not satisfed with experiments made upon cold blooded animals, the objection being raised that it was not safe to apply the conclusions drawn from them to the case of warm-blooded animals, employed the ears and wings of bats, 'in which (when one has acquired some art in quieting the animals with chloroform, or gentle management) nearly all of the phenomena of the circulation, as affected by the application of stimuli may be watched as deliberately as in the frog, and in some respects even more clearly.'

Following the suggestions of the author just quoted I applied to the spread wing of a bat fastened head downwards—a common way for the animal to suspend himself—upon a window pane, a red hot needle in several places, and observed with the aid of a pocket lens the effect upon the vessels distributed to the parts

This diagram shows the seemingly multiplied state of the vessels in the wing of a bat which has been punctured with a hot needle.—(After nature.)

irritated. Around the focus of the injury very soon after applying the needle, the wing seemed to take on vascularity. An endless multiplication of vessels appeared in the interphalangeal space. Upon applying it in another space, and watching it more closely from the first, the blood which was seen flowing rapidly in the arteries and veins of the transparent skinnymembrane, began to move more slowly, and in the vicinity of the burn to become temporarily stagnant. In the crossing and anastomozing vessels complete stagnation occurred close to the injury, and the coats of the vessels took on an aneurismal or varicose condition. At a little distance from the center of injury momentary contraction took place in the vessels, and then a somewhat permanent state of dilitation succeeded, allowing much more blood than usual to pass. No increase of vascular action was noticed in the opposite wing. While noticing the state of the burnt wing from day to day it was observed that the blood in the stagnant vessels began to move from time to time in the capillaries, gradually approaching the points of injury until every vessel except those which were charred, transmitted its usual fluid, and in streams not above the size of those that flowed before the harmony of the parts was disturbed. At the end of ten days the only difference to be observed in the two wings was the puckering effect of the cicatrices, and the little more wavy character of the principal artery and vein.

To repeat the experiment of Mr. Paget, if a fine needle be drawn across the companion artery and vein of a bat's wing three or four times without injuring them or the membrane that

This diagram represents the microscopic apearance of the capillaries of an inflamed tissue. In the lowest part of the picture nearest the focus of inflammation, the vessels are constringed: higher up they are seen dilated into pouches, and distorted. In the darkest portion the blood is stagnated; and effusions of serum and lymph have taken place into the intervascular spaces.—(From nature.)

covers them, they will in a short time, if the movement of the blood be watched, be perceived to gradually contract and close. But the blood will only remain stagnated a few minutes, for the vessels, after holding themselves contracted a short time, will begin to dilate moderately. and continue until they have acquired a larger calibre than they had before the stimulus was applied. The effect is most marked in the arteries, though the veins are not without the contractile force. If the needle be drawn across the vessels oftener and more forcibly, no contraction of any account ensues, and what does is quickly succeeded by dilitation. By this process so great a degree of local nervous exhaustion is produced that the vessels need a strong stimulus such as that of great heat, or a corrosive liquid, to excite them to contract and close. And when once contracted in this way, they need a long time to unload themselves of the agglutinated corpuscles, and permit the free flowing of blood through them.

Several important facts seem to be elicited by these experiments. First, that in local injuries the nerves are primarily effected, which in turn influences the muscular coats of the vessels, and the effects upon them are made manifest by the movement of the blood. As the vessels are contracting, the blood flows more slowly, or begins to oscillate, and if the stimulation be kept up, or increased, complete stagnation takes place in a greater or less number of vessels. The stagnation commences in the capillaries; and first in those which are least in the direct course from the artery to the vein; thence it extends, according to Mr. Wharton Jones, to the veins and to the arteries. In the focus of the inflammation there are most capillaries with stagnated blood in them, and the principal artery may be observed pulsating with every action of the heart; its current not being possible in the usual and most direct channels, is forced to pass off through collateral branches. The stagnated blood does not coagulate readily, but its corpuscles seem to change their hue, either through vital or chemical action or position, and take on a brilliant carmine appearance. The aggregation of the corpuscles in the contracted vessels, and the increase of number passing the dilated vessels, serve to increase the redness in an inflamed part.

Any one can satisfy himself that there is an accumulation of sanguinous fluid in and about the inflamed surface, and that the blood does not ordinarily coagulate in the part, by cutting into the inflamed tissues. Much more blood will be found to flow from the cut surfaces than from an incision into the healthy tissues in a corresponding place in an opposite part of the body."

## Figures and Tables

**Figure f1:**
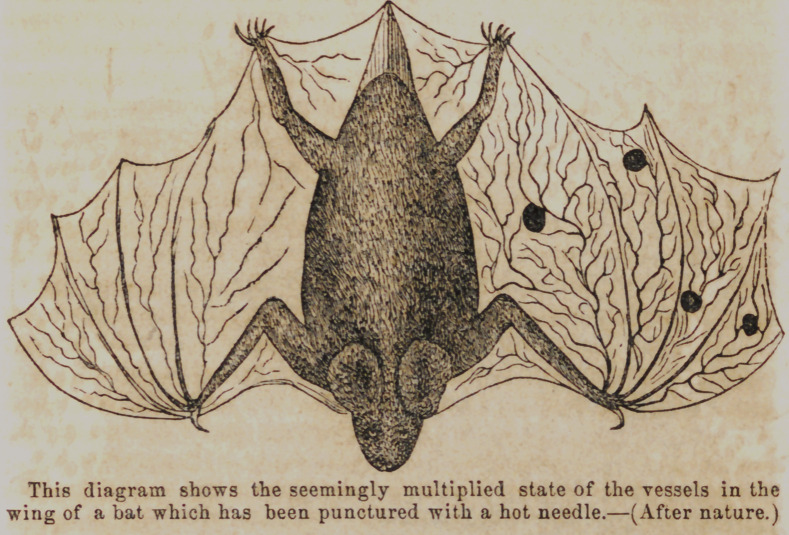


**Figure f2:**